# A Case of Complete Abscopal Response in High-Grade Pleiomorphic Sarcoma Treated with Radiotherapy Alone

**DOI:** 10.7759/cureus.821

**Published:** 2016-10-07

**Authors:** Andrew Orton, Jennifer Wright, Luke Buchmann, Lor Randall, Ying J Hitchcock

**Affiliations:** 1 Radiation Oncology, University of Utah School of Medicine, Huntsman Cancer Institute; 2 Medical Oncology, University of Utah School of Medicine, Huntsman Cancer Institute; 3 Department of Otolaryngology, University of Utah School of Medicine, Huntsman Cancer Institute; 4 Department of Orthopaedic Surgery/Sarcoma, University of Utah School of Medicine, Huntsman Cancer Institute

**Keywords:** abscopal, sarcoma, radiotherapy, head and neck cancer

## Abstract

Background: “Abscopal response” refers to the spontaneous involution of untreated metastatic disease following local primary tumor-directed therapy. We report a case of an abscopal response of untreated lung metastasis in a man with pleomorphic sarcoma of the head and neck treated with hypofractionated radiotherapy.

Methods: An inoperable pleomorphic sarcoma of the postauricular soft tissue was treated with 40 Gy of radiation in eight fractions. Untreated disease in the lungs was followed with CT scans.

Results: At the two-month post-treatment follow-up, clinical exam and restaging CT demonstrated complete primary tumor involution. Additionally, CT chest images showed a dramatic disease response in the untreated pulmonary disease, which progressed to complete and persistent clinical response at one-year post-treatment follow-up.

Conclusions: We report the first described case of a complete abscopal resolution of untreated lung metastases in a patient with a primary pleomorphic sarcoma of the head and neck treated with hypofractionated radiotherapy.

## Introduction

The term “abscopal” was originally coined by RH Mole in 1953 to describe damage to normal, non-dividing cells in subjects receiving total body irradiation [1]. Over the last 60 years, the term has evolved to describe the rare phenomenon of immune-mediated tumor regression at sites distant to the primary area of local therapy. There have been multiple previous reports of this effect in patients treated with radiation alone and, more commonly, radiation plus immunotherapy. It has been most common to seen this effect in patients with melanoma and renal cell carcinoma but has also been more infrequently reported in other disease histologies [2]. To date, the effect has not been reported in pleomorphic soft tissue sarcoma. We report a case of metastatic, high-grade pleomorphic soft tissue sarcoma of the postauricular region that showed dramatic involution of the patient’s untreated pulmonary metastases after radiotherapy was used for the tumor in the postauricular region.

Written permission was obtained from the patient to report this unusual outcome. Because the reported result is limited to one patient and does not meet the definition of “systemic investigation”, per Utah IRB SOP 308, the report was declared exempt from review.

## Case presentation

**Figure 1:**
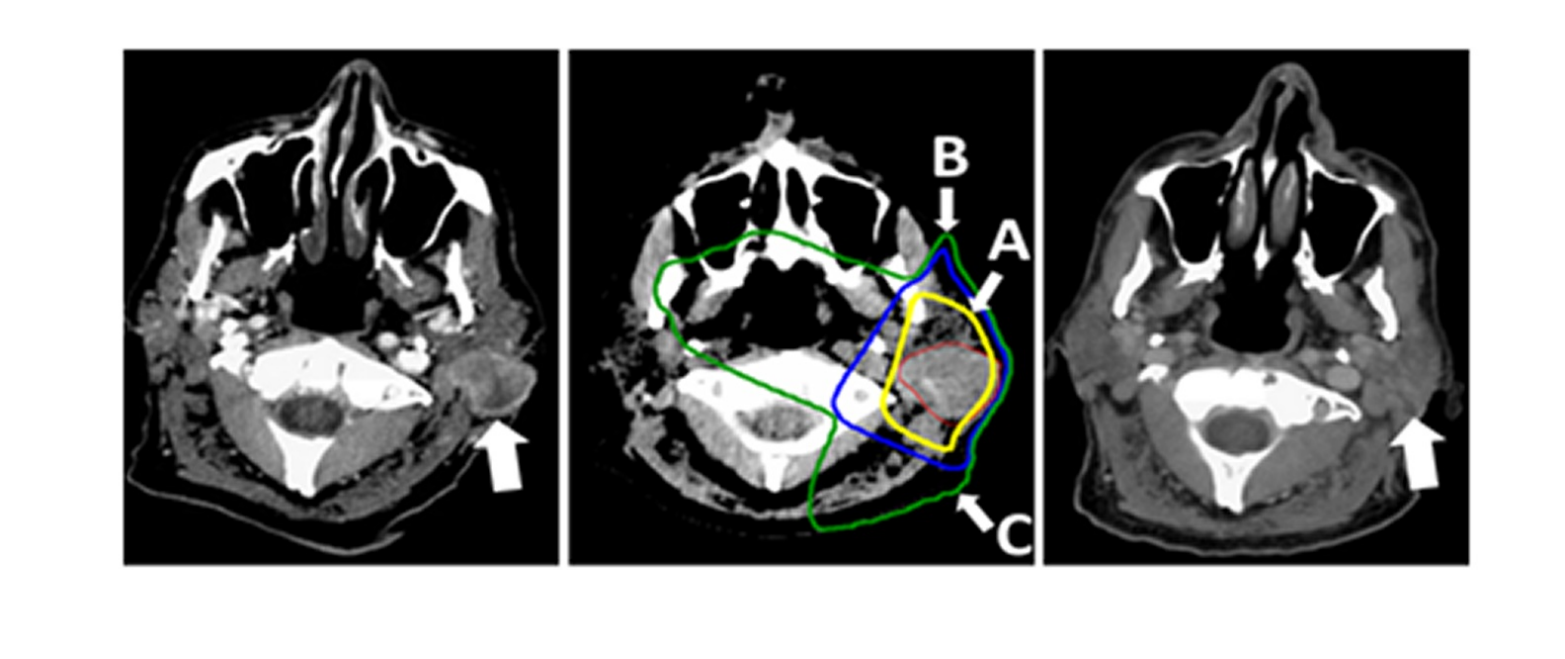
Axial Post-Contrast CT Images of the Primary Tumor Before and after Two Months Post-Treatment Axial post-contrast CT images of the primary tumor before (left, white arrow) and 2 months post treatment (right, white arrow). The involution in tumor size was so dramatic the radiology report was read as “status/post resection of the mass between the left sternocleidomastoid and parotid gland,” even though no surgery had been done. A representative image of the radiotherapy plan is also shown (center panel). 95% (A), 50% (B) and 30% (C) isodose lines are shown.

The patient is an 84-year-old man who was diagnosed in the summer of 2013 with pleomorphic soft tissue sarcoma of the scalp. The lesion was multiply recurrent, and he underwent three resections between 2013 and 2015 with repeat recurrences in the scalp despite negative margins. In February of 2015, the patient noted an enlarging mass just behind the pinna of his left ear. A CT scan documented a 2 cm mass that was intimately related to the parotid gland (Figure 1, left panel).

**Figure 2:**
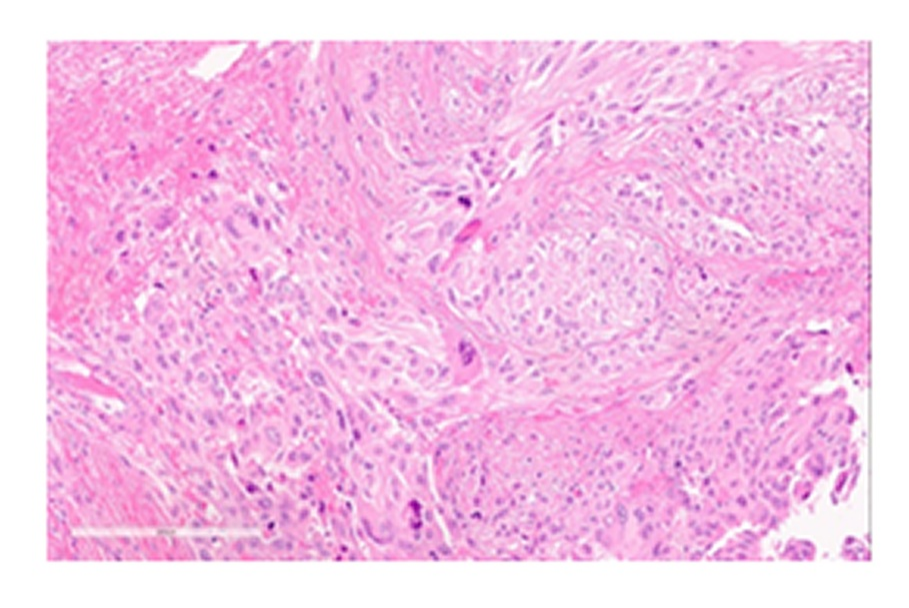
A Representative H+E Section of the Tumor Before Radiotherapy A representative H+E section of the tumor before radiotherapy. The neoplastic cells are negative for S100, HMB 45, CD34, CD31 and CAM 5.2. The overall morphologic features and immunoprofile are consistent with high-grade undifferentiated pleomorphic sarcoma. No significant infiltration of lymphocytes or macrophages is noted.

A fine needle aspiration was obtained of the mass that was thought to be most consistent with a recurrence of the patient's known sarcoma. A confirmatory biopsy was then done, which was reported as high-grade malignant spindle cell neoplasm consistent with undifferentiated pleomorphic sarcoma. The lesion was noted to be highly mitotically active with 32 mitoses per 10 HPF (Figure 2).

**Figure 3:**
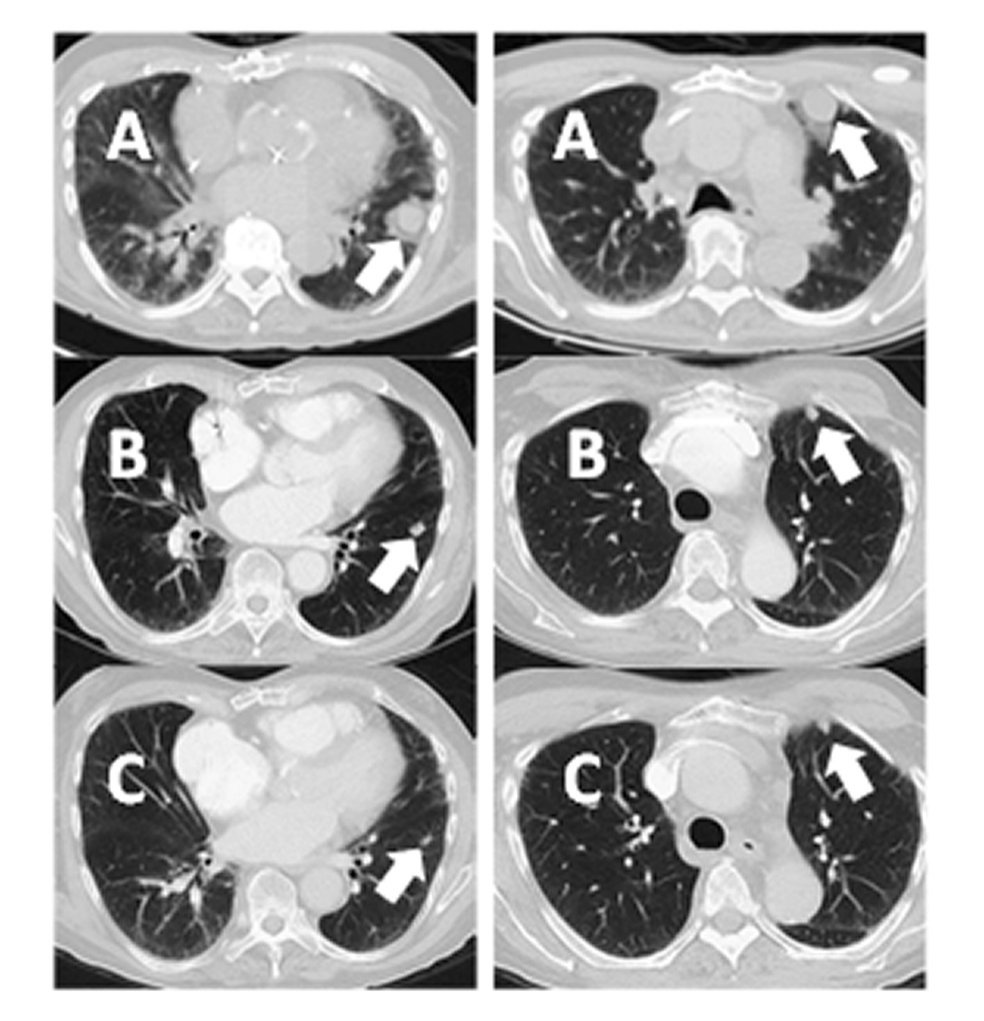
A Representative Series of Images Demonstrating the Abscopal Response Noted in the Untreated Lung Images shown are pretreatment (A) As well as post-treatment at two months (B) and four months (C). The other two lesions (not shown) showed a similar response, and all pulmonary disease had completely regressed at ten months follow-up.

A PET/CT was obtained, given the high-risk features of his disease, which documented three hypermetabolic nodules involving the lungs consistent with metastatic disease (Figure 3, top panel).

At the time of his surgical consultation with otolaryngology, the patient declined surgery, citing his age and medical comorbidities, which included significant heart disease. Therefore, he was referred to radiation oncology for palliative radiotherapy to the postauricular lesion.

He received a course of 3D conformal external beam radiation to a dose of 40 Gy in eight fractions using electrons (Figure 1, center panel). Treatment was tolerated very well with toxicity limited to only Grade 1 skin erythema.

Following completion of his radiotherapy, the patient was seen by medical oncology to discuss chemotherapy options to address his lung disease. Given his medical comorbidities, and low response rate with this histology, systemic therapy was not offered.

**Figure 4:**
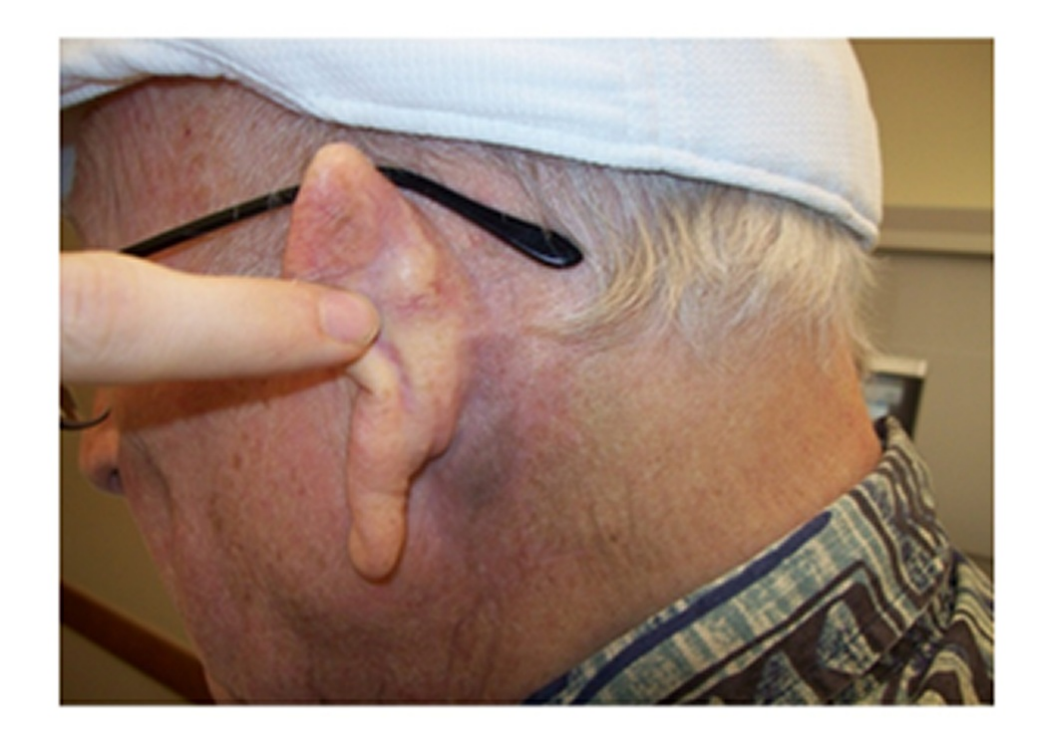
Two Months Post-Treatment At two months post-treatment, minimal skin changes over the treated site could be noticed. There were no subjective changes in hearing acuity or salivary gland function.

At two his two-month post-treatment follow-up, clinical exam and restaging CT neck soft tissue demonstrated complete tumor response without residual disease in the irradiated left postauricular region. Computed tomography chest images showed a dramatic disease response in the untreated pulmonary disease (Figure 1, right panel; Figure 2, middle and bottom panels). The skin over the posterior ear, which had been threatened by tumor eruption, showed only faint Grade 1 hyperpigmentation (Figure 4).

An otoscopic evaluation revealed a normal-appearing external auditory canal. Subjectively, the patient's follow-up at four, seven, 10, and 13-months again documented complete and persistent resolution of both the primary tumor and of the untreated lung metastases.

## Discussion

We have reported a case of a dramatic abscopal tumor response in a patient treated with radiotherapy alone for high-grade pleomorphic sarcoma of the head and neck. This effect, not infrequently noted in other disease sites such as melanoma and renal cell carcinoma, has not been reported in pleomorphic sarcoma, a histology historically thought to have some degree of radioresistance. There have also been previous reports of abscopal regression in other tumors of other unusual histology, including hepatocellular carcinoma [3].

Recent laboratory data has significantly added to our understanding of the mechanism of the abscopal effect; ionizing radiation induces mitotic catastrophe and the resultant antigen release primes the native immune system to recognize and attack distant metastatic tumor cells [2]. Furthermore, we know that CD-8 T cells and macrophages are an essential component of the effect in humans [4-5]. Considerable interest is now directed at harnessing this effect in the clinic, using newly approved immune-modulating agents.

There are several unanswered questions that are being investigated to determine how to induce the native immune system to recognize and attack metastatic tumor cells, including the optimal fractionation size of local radiotherapy to induce tumor antigen expression, the timing of delivery of radiotherapy in conjunction with immunotherapy [6-7], and which drug therapies show the best promise in priming the immune system [8-10].

## Conclusions

Achieving an immune-mediated systemic response after localized radiotherapy in the clinic is more than a “gee-whiz” event; retrospective data have shown that patients who achieve an abscopal response have superior overall survival than those who do not. This case is exciting because it suggests that high-grade pleomorphic sarcoma may, like melanoma and renal cell carcinoma, have exploitable immune-mediated pathways that can extend both quality of life and overall survival in patients with metastatic disease. This case is hypothesis generating, and may suggest a role for hypofractionated radioablative treatment of the primary disease site in conjunction with immunotherapy.
